# Abnormalities in Glutamate Metabolism and Excitotoxicity in the Retinal Diseases

**DOI:** 10.1155/2013/528940

**Published:** 2013-12-09

**Authors:** Makoto Ishikawa

**Affiliations:** Department of Ophthalmology, Akita Graduate University Faculty of Medicine, 1-1-1 Hondo, Akita 010-8543, Japan

## Abstract

In the physiological condition, glutamate acts as an excitatory neurotransmitter in the retina. However, excessive glutamate can be toxic to retinal neurons by overstimulation of the glutamate receptors. Glutamate excess is primarily attributed to perturbation in the homeostasis of the glutamate metabolism. Major pathway of glutamate metabolism consists of glutamate uptake by glutamate transporters followed by enzymatic conversion of glutamate to nontoxic glutamine by glutamine synthetase. Glutamate metabolism requires energy supply, and the energy loss inhibits the functions of both glutamate transporters and glutamine synthetase. In this review, we describe the present knowledge concerning the retinal glutamate metabolism under the physiological and pathological conditions.

## 1. Introduction

**Figure 1:**
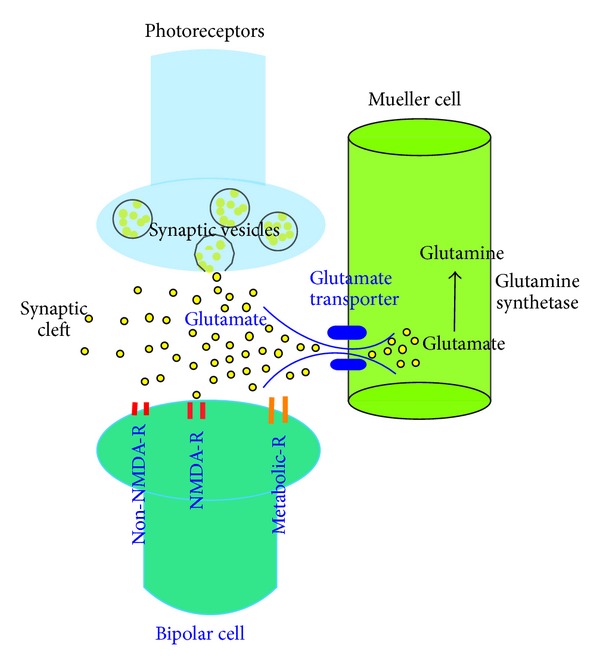
Glutamatergic synaptic transduction and uptake and metabolism of glutamate in the Müller cell. The photoreceptor cell synthesizes glutamate, which is continuously released during darkness. Glutamate released from the synaptic terminal reaches to the postsynaptic receptors of bipolar cell dendrites, then is promptly taken up from the synaptic cleft. The glutamate transporters are the predominant mechanism for uptake of glutamate in the Müller cell, and maintain the proper concentration of this potentially excitotoxic amino acid. Glutamate transported into the Müller cell is degraded to glutamine by glutamine synthetase.

Glutamate is the most prevalent neurotransmitter in the visual pathway including the retina [1–3]. After release from the presynaptic neural terminal, glutamate binds to the postsynaptic glutamate receptors, inducing the influx of Na^+^ and Ca^2+^, resulting in membrane depolarization. When glutamate is in excess, it can become toxic to retinal neurons by overstimulation of the glutamate receptors [4–7]. In as early as 1957, Lucas and Newhouse [4] reported that subcutaneous administration of sodium L-glutamate induced necrosis in the inner retina of albino mice within a few hours of injection, indicating that high concentrations of glutamate cause retinal cell death. Therefore, efficient removal of glutamate from the extracellular space is critical for maintenance of retinal function and preventing the retinal neurons against glutamate toxicity (Figure 1).

Glutamate is metabolized by two major processes: its uptake and the subsequent enzymatic degradation. Derouiche and Rauen (1995) [8] have shown that glutamate is degraded into nontoxic glutamine by glutamine synthetase, following uptake by major glutamate transporter, GLAST, into Müller cells. A prerequisite for an effective glutamate-glutamine cycle in glial cells is the regulated coordination between glutamate uptake and glutamate degradation [9].

Although glutamate is considered as a potent exotoxin, exogenous glutamate is only weakly toxic to the retina when glutamate transporters on Müller glial cells are operational [10]. When glutamate transporter is pharmacologically blocked, inner retinal neurons are exposed by a higher amount of endogenous glutamate, resulting in severe excitotoxic degeneration [10]. These observations suggest that glutamate is neurotoxic when the uptake system is impaired rather than when the release is excessive.

The first part of this article surveys physiology of glutamate metabolism with particular interest in glutamate transporters (GLAST, GLT-1, EAAC-1), glutamine synthetase, and energy supply. The second part describes excitotoxic retinal degeneration induced by abnormalities of glutamate metabolism in the retinal ischemia, glaucoma, and diabetic retinopathy.

## 2. Glutamate Metabolism in the Physiological Condition

### 2.1. Glutamate Uptake by Glutamate Transporters

All neuronal and glial cells in the retina express high-affinity glutamate transporters [9]. At least five glutamate transporters have been cloned: GLAST (EAAT1), GLT-1 (EAAT2) [11, 12], EAAC-1 (EAAT3) [13], EAAT4 [14], and EAAT5 [15]. In the retina, four out of the five known EAATs have been described. Immunohistochemical analysis localize the distributions of these glutamate transporters, respectively, [16, 17]. GLAST is distributed in the Müller glial cells. GLT-1 and EAAT5 are colocalized in the photoreceptor cells and the bipolar cells. EAAC-1 (EAAT3) is localized in the horizontal cells, ganglion cells, and some amacrine cells.

Since glutamate is a potent neurotoxin, the functional role of glutamate transporters is critical to prevent the retinal excitotoxicity. Izumi et al. [10] reported the pharmacological blockade of glutamate transporters using TBOA (DL-threo-!-benzyloxyaspartate) [18–20]. TBOA is an inhibitor of all types of glutamate transporters, and does not evoke currents in neurons or glial cells. Izumi et al. [10] used TBOA alone and in combination with exogenous glutamate to examine the role of glial glutamate transporters in excitotoxic retinal degeneration. In the presence of TBOA, the potency of glutamate as a neurotoxin is greatly enhanced. Surprisingly, TBOA alone is neurotoxic, and the toxicity is inhibited by a combination of ionotropic NMDA and non-NMDA receptor antagonists, suggesting that the damage is excitotoxicity. Furthermore, TBOA-induced neuronal damage is inhibited by the glutamate release inhibitor, riluzole, suggesting that the damage is mediated by endogenous glutamate, rather than resulted from a direct action of TBOA. These results strongly suggest that the neurotoxic actions of TBOA result from blocking glutamate transport, and pathological conditions or treatments that impair glial glutamate transport greatly augment the toxic effects of endogenous glutamate.

Recently, antisense knockout techniques or the construction of transgenic and gene knockout mouse models represent the successful approach to achieving suppression or elimination of a genetic message of specific glutamate transporters. In this section, we describe the characterization of these three glutamate transporter precisely based on the experimental results mainly obtained by gene engineering.

#### 2.1.1. GLAST

GLAST is the prominent glutamate transporter in the retina, and mainly expressed in the Müller cells [1, 8, 21–24]. In the mouse Müller cells, glutamate removal by GLAST is calculated as 50% among the retinal glutamate transporters [25]. Glutamate uptake via GLAST is accompanied by cotransport of three Na^+^ and one H^+^, and the counter-transport of one K^+^ at each glutamate transport. Sodium ions are extruded, at least in part, by Na^+^/K^+^-adenosine triphosphate (ATP) ase in a reaction that consumes ATP.

To elucidate the role of GLAST in the regulation of retinal function, Harada et al. [26] developed GLAST-deficient mice, and revealed reduction of the scotopic ERG b-wave, indicating the reduction of glutamate-mediated neurotransmission activity in the retina. After induction of the retinal ischemia by increasing intraocular pressure above systolic pressure for 60 min, more severe excitotoxic degeneration is found in GLAST-deficient mice than in wild-type, indicating that GLAST is neuroprotective against ischemia [27].

Barnett and Pow [28] injected intravitreally antisense oligonucleotides to GLAST into rat eyes. Although a marked reduction of GLAST activity was detected, the retinas displayed no evidence of excitotoxic neuronal degeneration, and the distribution of glutamate was unaffected by antisense treatment. Significant inhibition in GLAST function was apparent 5 days after injection of antisense oligonucleotide and was sustained for at least 20 days. The observed lack of neuronal degeneration suggests that reduced glutamate uptake into the Müller cells does not cause excitotoxic tissue damage.

However, Harada et al. [29] examined the long-term effect (32 weeks) of GLAST deficiency on the retinal morphology during postnatal development *in vivo*, and revealed spontaneous loss of retinal ganglion cell (RGC) and optic nerve degeneration without elevated intraocular pressure (IOP). In GLAST-deficient mice, administration of glutamate receptor antagonist prevented RGC loss. These findings suggest that GLAST is necessary to prevent retinal excitotoxicity.

Taken together, the glutamate removal by glutamate transportes is prerequisite for the maintenance of normal retinal transmission, and preventive against excitotoxicity.

#### 2.1.2. GLT-1

Unlike in the central nervous system [30], it is considered that GLT-1 does not play a predominant role in the physiological glutamate transmission in the retina because antisense GLT-1-knockout mice exhibit almost normal retinal function [24, 30]. However, other researchers reported that treatment with antisense oligonucleotides against GLT-1 increased vitreal glutamate levels leading to ganglion cell death in the rat retina [31], and the retinal damage induced by ischemia was exacerbated in GLT-1 deficient mice [26]. Harada et al. [29] tried to examine the long-term effect of GLT-1 deficiency on the retinal morphology during postnatal development *in vivo*. However, almost all of GLT-1^−/−^mice died within 3 weeks. There seems to be no difference in RGC number between GLT-1^+/−^mice and wild-type mice.

Despite these contradictory findings, the involvement of GLT-1 of the retina in the homeostasis of glutamate cannot be excluded.

#### 2.1.3. Splice Variants of Glutamate Transporters

Splice variant is a result of alternative splicing of pre-mRNA where the exons are reattached in a different manner to produce different mRNAs [32]. Alternative splicing occurs in 95% of multiexonic genes [33]. Splice variants are translated from alternatively spliced mRNAs that contain diversities in amino acid sequence or biological properties.

*(a) GLAST*. The presence of three types of splice variants of GLAST (GLAST1a, GLAST1b, and GLAST1c) has been reported. GLAST1a and GLAST1b lack exon 3 and 9, respectively [34, 35]. GLAST1a preferentially located in the endfeet of the Müller cells. The localization of GLAST1a is different from normally expressed GLAST, which is entirely expressed in the Müller cell body [34]. However, the clarification of the precise role of GLAST1a in the retina is still remained. GLAST1b is expressed at low levels in neurons in the normal brain, while expression levels rise dramatically in neurons after a hypoxic insult, indicating that expression is regulated to maintain the homeostasis of the glutamate concentration [36]. GLAST1c lacks both exon 5 and 6 and coded for a 430 amino acid protein, [37]. GLAST1c is present in multiple species and is widely expressed by astroglial cells and oligodendrocytes in the brains of various mammalian species as well as in the optic nerve and the retinas of human. Similarities between GLAST1c and a functional prototypic transporter may support the speculation that GLAST1c is a functional glutamate transporter, and possibly represents a primitive form of GLAST. However, the precise localization of GLAST1c in the retina has not been identified.

*(b) GLT-1.* GLT-1 (EAAT2) exists in several distinct forms, including the originally described form (GLT-1a), along with GLT-1b (also called GLT1v) and GLT1c [38]. GLT-1a appears to be associated mainly with a population of amacrine cells, whereas GLT-1b is associated with cone photoreceptors, subpopulations of bipolar cells, and astrocytes [39, 40]. GLT-1c is normally only expressed by the photoreceptors in the mammalian retina [38]. In the rat, GLT1c is colocalized with GLT1b in cone photoreceptors. GLT1c expression is developmentally regulated, only appearing at around postnatal day 7 in the rat retina, when photoreceptors first exhibit a dark current [41]. In the normal eyes of humans and rats, GLT-1c was expressed only in photoreceptors. In glaucoma, there was an apparent increase in expression of GLT-1c in retinal ganglion cells, including occasional displaced ganglion cells. Although the precise role of GLT-1c still remains to be elucidated, upregulation of GLT-1c might be an attempt to adapt to the pathological condition such as glaucoma.

Based on these findings, transcriptional regulation and mRNA splicing causing differential expression of GLAST or GLT-1 may affect glutamate transport and may provide an complicated mechanism for glutamate uptake.

*(c) EAAC-1*. EAAT3 is also known as excitatory amino acid carrier 1 (EAAC-1). EAAC-1 localizes in the synaptic layers (the outer and inner plexiform layers), horizontal cells, subgroups of amacrine cell, and the ganglion cell. In addition, immunoreactivity reveals the presence of EAAC-1-positive amacrine cells distant from the synaptic sites. Considering the location, EAAC-1 may regulate glutamate uptake in different manner both near and well away from synaptic sites. However, knockdown of the expression of GLAST or GLT-1 in rats using antisense oligonucleotides increased the extracellular glutamate concentration, whereas EAAC-1 knockdown mice showed no increase in extracellular glutamate [42]. These findings indicate that glutamate uptake is not a major role of EAAC1. EAAC-1 can transport cysteine significantly higher than GLAST or GLT-1 [43] and contribute to generate glutathione. Partial knock-down of EAAC-1 decreases the neuronal glutathione contents and increases oxidant levels [44]. These results indicate that EAAC-1 is responsible for the metabolism of glutathione, which plays a critical role as an antioxidant.

### 2.2. Glutamate Degradation by Glutamine Synthetase

Glutamine synthetase is the only enzyme to synthesize glutamine, and plays an important role in glutamate detoxification [45]. Glutamine synthetase seems critical for the cell survival, because the deficiency in the glutamine synthetase gene induces early embryonic death [46].

In the retina, glutamine synthetase is specifically localized in the Müller cells [47], and catalyzes the ATP-dependent condensation of glutamate [48]. In salamander retina, glutamine synthesis stimulates glial glycolysis to match energy demands [49]. Additionally, glutamine synthetase activity is most prominent in the presence of high levels of ammonia, and is more limited in the presence of low levels of ammonia [49]. Immunohistochemistry revealed that inhibition of glutamine synthetase by d,l-methionine,l-sulfoximine (MSO) caused a dramatic increase in the glutamate level in the Müller cells, and subsequently the rapid loss of the glutamate content of the photoreceptor cells, bipolar cells, and ganglion cells [8]. Barnett et al. [50] injected MSO intraocularly in Wistar rats, and revealed prompt suppression of the scotopic ERG b-wave, indicating the reduction of glutamatergic neurotransmission activity in the retina. These results also indicate that inhibition of glutamine synthetase may rapidly impair the retinal response to light.

### 2.3. Importance of Energy Supply in Glutamate Metabolism

Glutamate uptake can be influenced by changes in cellular energy levels [51, 52]. ATP is the usable form of chemical energy for the central nervous system including the retina. ATP is provided by two different sources: oxidative metabolism and glycolysis. The generation of ATP through oxidative metabolism (oxidative ATP) is selectively blocked by oxygen deprivation. The generation of ATP through glycolysis (glycolytic ATP) is not blocked by glucose deprivation, as long as glycogen stores are available for use. Iodoacetate (IA) inhibits both oxidative and glycolytic ATP generation.

Oxygen deprivation produces little morphological change and does not induce glutamate-induced excitotoxicity. In contrast, inhibition of glycolysis by IA produced severe neuronal damage. The neuronal damage produced by IA was inhibited by pyruvate, a substrate that sustains oxidative energy pathways. In the presence of IA plus pyruvate, glutamate became neurotoxic at low concentrations through activation of non-NMDA receptors [53]. These results indicate that glycolytic energy metabolism plays a critical role in sustaining ionic balances that one required for Müller cell glutamate uptake, and glial uptake helps to prevent glutamate-mediated excitotoxicity.

Even in the presence of normal energy metabolism, however, ATP levels will be depressed when ATP consumption exceeds production. Thus, excessive neuronal activity and energy demands may acutely reduce ATP levels. Energy failure increases vulnerabilities of retinal neurons to excitotoxicity, because the critical steps in glutamate metabolism are ATP-dependent. In this section, we give an overview of the relations between the energy supply and the glutamate metabolism.

#### 2.3.1. Glutamate Transporters

Although glutamate uptake by glutamate transporters does not require ATP consumption, Glutamate transporte is the Na^+^-dependent glutamate transporters, which utilize ionic gradients of Na^+^, K^+^, and H^+^ to drive glutamate transport against the concentration gradient. Since the ionic gradients are maintained by the sodium pump, they are dependent upon ATP production. When ATP levels drop, increased extracellular K^+^ and reduced extracellular Na^+^ result in a neurotoxic release of glutamate, and are attributed to reversed operation of glutamate transporters [54]. Reversal of the glutamate transporter is considered to facilitate the increase of extracellular glutamate concentration to excitotoxic levels. These findings also indicate that the glutamate transporter may transport glutamate in either direction depending on the ionic gradient across the plasma membrane.

#### 2.3.2. Glutamine Synthetase

Degradation of glutamate by glutamine synthetase also consumes ATP molecule as in the following reaction:

(1) Glutamate+NH4++ATP   →  glutamine+ADP+Pi+H+

in the presence of manganese or magnesium. Therefore, the suppression of intracellular ATP causes a decrease in glutamine synthetase activity.

GLAST and glutamine synthetase are the primary role players that transport glutamate into the Müller cell and convert it into glutamine. Jablonski et al. [55] reported the genetic regulation of both genes *Slc1a3 *and* Glul* using an array of 75 recombinant inbred strains of mice. *Slc1a3 *and* Glul* encode GLAST and glutamine synthetase, respectively. Interestingly, despite their independent regulation, gene ontology analysis of tightly correlated genes reveals that the enriched and statistically significant molecular function categories of both directed acyclic graphs have substantial overlap, indicating that the shared functions of the correlates of *Slc1a3* and *Glul* include production and usage of adenosine triphosphate (ATP). These results indicate that ATP depletion may induce excitotoxicity via downreguration of *Slc1a3 and Glul. *Consistently, it has been reported that energy deprivation decreases glutamate uptake within 2-3 min [56].

In addition to the suppression on the glutamate transporters and glutamine synthetase, ATP depletion also induces the failures in membranous Na^+^/K^+^ pump maintaining the resting membrane potential, or in Ca^2+^ extrusion. Intracellular calcium increase leads to mitochondrial impairment, further accelerating ATP depletion [57]. Recently, Nguyen et al. propose a vicious cycle involving excitotoxicity, oxidative stress, and mitochondrial dynamics. Oxidative stress produced by mitochondrial impairment leads to the upregulation of the NMDA receptors, and exaggerates the excitotoxicity [58].

## 3. Abnormalities of Glutamate Metabolism

Excitotoxicity has been considered to be involved in several ocular pathologies including ischemia induced by retinal or choroidal vessel occlusion, glaucoma, and diabetic retinopathy [1–7]. Inhibition of retinal degeneration afforded by administration with glutamate receptor antagonists supports this hypothesis [1, 3, 8, 9, 59, 60].

Excitotoxic cell death does not always result from excess of glutamate. Extracellular levels of glutamate achieved during retinal ischemia [61–63] may not be sufficient to induce neuronal damage under normal conditions [64]. This suggests that clearance of glutamate is important in preventing retinal excitotoxicity in response to glutamate. In support of this, it has been shown that retinas of GLAST deficiency mice are extremely sensitive to the ischemic insults [26].

In this section, we describe the abnormalities of glutamate mechanisms in retinal ischemia, glaucoma, and diabetic retinopathy, respectively.

### 3.1. Retinal Ischemia

In general, ischemia means the pathological conditions with a restriction in blood flow, causing an inadequate supply of oxygen and glucose needed for cellular metabolism. In consequence, the prolonged retinal ischemia induces irreversible morphological and functional changes. For example, the experimental obstruction of the central retinal artery of old, atherosclerotic, hypertensive rhesus monkeys induced no remarkable changes within 97 min, while the longer the arterial obstruction, the more extensive the retinal damage [65].

Acute retinal ischemia induces irreversible damages as the consequence of ATP depletion [66–68]. ATP depletion lowers the function of Na^+^-K^+^-ATP pump, resulting in influx of Na^+^, Cl^−^, and water, which can cause hypo-osmotic swelling of cell organelles and their dysfunction [69]. Reperfusion after ischemia has been known to induce more exaggerated retinal degeneration. The cessation of oxygen and nutrients supply during ischemia increases the susceptibility of the retina for inflammation [70] or the oxidative stress induced by the restoration of blood circulation. Excitotoxicity is considered as one of the essential elements to trigger the above-mentioned ischemia/reperfusion degeneration [71].

#### 3.1.1. Glutamate Transporters

In the retinal ischemia, the expressional and functional changes of glutamate transporters have been reported. Barnett et al. [72] have reported that GLAST functions are reduced after retinal ischemia was induced by the central retinal artery occlusion for 60 min, while the GLAST expression appears to be normal. Decrease in glutamate uptake by GLAST may induce diffusion of glutamate into vitreous cavity or anterior chambers. In consistence, it is reported that glutamate concentration significantly increased in aqueous humor sample obtained from patients with retinal artery obstruction [73].

The ischemia/reperfusion model revealed a marked increase in GLAST mRNA expression in the inner retina [74]. By contrast, Barnett and Grozdanic [75] reported that the GLAST function recovers rapidly upon reperfusion, and suggest that the loss of transporter function might be due to acute metabolic changes induced by the ischemia [76–78], rather than rapid downregulation of transporter gene expression.

Recently, Russo et al. [79] evaluated the expression of GLAST and GLT-1 in a rat ischemia/reperfusion model. They reported that there were no remarkable changes in GLAST expression, while modulation of GLT-1 expression was observed in the isolated retinal synaptosomes. These results support a role for GLT-1 in glutamate accumulation observed in the retina following an ischemic event.

Despite of discrepancies, these findings suggest that glutamate transporters play an important role in the regulation of extracellular glutamate concentration under ischemic conditions.

#### 3.1.2. Glutamine Synthetase

As glutamine synthetase consumes ATP to convert glutamate to glutamine [80], it seems plausible that ATP suppression in the ischemic retina [21] promptly induces the impairment of delegation of glutamate, resulting in the elevation of the extracellular concentration of glutamate. However, glutamine synthetase activity was relatively well preserved during 60 minutes of simulated acute retinal ischemia induced by deprivation of both oxygen and glucose using *ex vivo* rat retinal preparation [21]. Such results indicate that the retina can efficiently generate ATP from glycolysis despite of the deprivation of oxygen and glucose [81]. In ischemia/reperfusion rat eyes, it actually takes several days to induce a significant depression of glutamine synthetase after the onset of the retinal ischemia [82–84].

The response of the retina to a postischemic reperfusion phase seems to depend upon the intensity of the ischemic stress. The other researcher reported that the expression in glutamine synthetase in Müller cells increased at 6 hours after ischemia reached its peak at 24 hours and decreased on day 14 compared with normal level in the ischemia/reperfusion model [85].

It is considered that glutamine synthetase activity may be upregulated for a short period after onset of ischemia to protect retinal neurons against excitotoxicity. As the rates of glycolysis decrease according to the depletion of glycogen storage, an intracellular ATP level falls, causing inhibition of glutamine synthetase. In addition, it is plausible that ischemia induced upregulation of protein kinase C delta, resulting in the downregulation of glutamine synthetase [86].

### 3.2. Glaucoma

Glaucoma is characterized by progressive and accelerated loss of retinal ganglion cells and their axons. The prominent pathological finding in glaucoma is the apoptotic cell death of the RGC [87]. However, the pathogenesis of apoptotic RGC death in glaucoma has not been clarified. It is hypothesized that glutamate-mediated excitotoxicity may contribute to pathogenesis of glaucoma [88]. Although an elevation of glutamate concentration in the vitreous humor was reported [89, 90], the following studies could not reproduce these results [91–94]. However, an elevation in the glutamate level in the vitreous humor is not necessary to induce excitotoxicity in the experimental animals or humans with glaucoma [95, 96]. It is because glutamate increase is likely to occur only in localized areas of the retina or optic nerve at any one time during glaucomatous neurodegeneration. If this is true, abnormalities in the glutamate metabolism result in excitotoxic damage to the RGC and contribute to the pathophysiology of glaucoma.

#### 3.2.1. Glutamate Transporters

Glutamate transporters are critical for maintaining optimal extracellular concentrations of glutamate [4, 5, 59, 97, 98]. Since glutamate transport is the only mechanism for removing glutamate from the extracellular fluid, it is hypothesized that functional impairment of glutamate transporters may play a major role in excitotoxicity and contribute to the pathogenesis of glaucoma [99, 100]. Changes of GLAST, GLT-1, and its splice variant, and ECCA-1 have been reported in glaucoma.

*GLAST*. GLAST is the major glutamate transporter expressed in retinal Müller cells [48]. However, the expressional changes of GLAST in glaucoma are still controversial. Some studies have shown that GLAST expression diminishes [99–101] or remains stable [102] in experimental glaucoma, whereas others have reported an increased expression [103]. In this section, I will overview the role of GLAST in the pressure-dependent and the pressure-independent glaucoma models.

*(a) Pressure-Dependent Glaucomatous Changes with GLAST*. It is generally recognized that elevated intraocular pressure is the most significant risk factor for accelerated ganglion cell death in glaucoma. However, the mechanism of cellular damage caused by elevated IOP is still unknown.

It has been reported that GLAST expression is reduced in experimental glaucoma models of rat [100] and mouse [101], as well as in glaucoma patients [99]. By contrast, there is another report that GLAST expression increased time-dependently in a rat glaucoma model [103]. These discrepancies might be mainly caused by the differences in the antibodies and glaucoma model.

**Figure 2:**
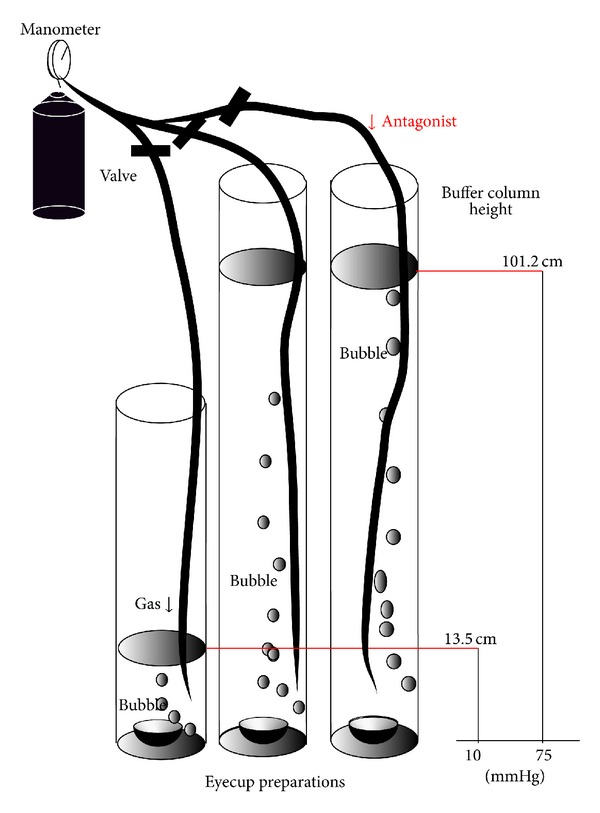
Outline of the present experiments. Eyecups preparations were sunken to the bottom of a glass cylinder with different heights. Each glass cylinder was filled with incubation buffer at 30°C for 24 hours. The buffer was bubbled with 95% O_2_-5% CO_2_ Hydrostatic pressure at the bottom of the cylinder was calculated to be 10 mmHg and 75 mmHg when a CSF was added to a height of 13.5 cm and 101.2 cm, respectively. Glutamate receptor antagonists or agonists were added to the buffer during some experiments.

**Figure 3:**
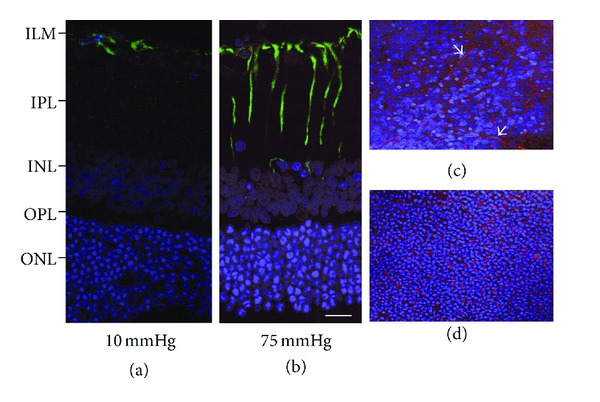
Immunofluorescent localization of glial fibrillary acidic protein by confocal fluorescent microscopy. (a) GFAP expression is recognized as FITC fluorescence, and localized in the Müller cells endfeet of (arrow) in a normal retina (10 mmHg). (b) Apparent fluorescent reaction is recognized in the Müller cell body (arrowheads) and the Müller cells endfeet (arrow) at 75 mmHg. (c) and (d) Tangential view of the GFAP expression at the level of the inner nuclear layer (c) and the outer nuclear layer (d). GFAP-stained Müller cell bodies were recognized as (rhodamine-stained) reddish dots among the DAPI- (4′,6-diamidino-2-phenylindole dihydrochloride) stained nuclei. Note the obliquely running Müller cell process (arrows). Figures (a) to (d) are in the same magnification. Figures (a) to (d) are in the same magnification. Bar = 20 *μ*m.

We recently developed a rat *ex vivo* hydropressure model (Figure 2) to examine the expressional changes of GLAST induced by elevated pressure (75 mmHg) for 24 hours [104]. Such acute high pressures can induce retinal ischemia clinically and in *in vivo* glaucoma models [105–107]. The *ex vivo* hydrostatic pressure model takes advantages to exclude the effects of ischemia, and could investigate direct the effects of pressure-induced retinal injury on glutamate metabolism (Figure 3). In this acute model, Western blot and real-time RT-PCR analyses revealed that 75 mmHg pressure inhibited GLAST expression [104].

*(b) Pressure-Independent Glaucomatous Changes with GLAST*. The population-based study revealed that the normal-tension glaucoma is the most prevalent form of glaucoma in Japan. Moreover, in some glaucoma patients, significant IOP reduction does not prevent the progression of the disease. Harada et al. [29] show that GLAST deficient mice demonstrate spontaneous ganglion cell death and optic nerve degeneration without elevated IOP. In GLAST-deficient mice, administration of glutamate receptor blocker prevented RGC loss, indicating that GLAST is necessary to prevent excitotoxic retinal damage. Additionally, GLAST maintains the glutathione levels in Müller cells by transporting glutamate, the substrate for glutathione synthesis, into the cells. Glutathione has strong antioxidative properties. Taken together, GLAST deficiency leads to RGC degeneration caused by both excitotoxicity and oxidative stress.

*GLT-1*. GLT-1 is one of the major glutamate transporters along with GLAST, and is found only in cones and various types of bipolar cells. The characteristic localization of GLT-1 on bipolar cells in the vicinity of the ganglion cell implies that GLT-1 may regulate glutamate concentration around ganglion cell synapse [100]. It is because the specific subtype of ganglion cells is known to degenerate during glaucoma [108].

GLT-1 is reported to be down-regulated in glaucomatous eyes in rats [100] and mice [101]. However, Park et al. [102] reported that GLT-1 was expressed in cone photoreceptors and some cone bipolar cells and the levels of expression were significantly increased in *in vivo* rat glaucoma model. In contrast, GLAST expression, which occurred in Müller cells, the main retinal glial cells, remained stable during the experimental period. These results suggest that integrity of GLT-1 may be a prerequisite for the maintenance of glutamate homeostasis in the retina undergoing glaucoma [109].

Additionally, one of the splice variants of GLT-1, GLT-1c, showed clear response against the IOP elevation. In normal eyes of humans and rats, GLT-1c was expressed only in photoreceptors. In glaucoma, there was additional preferential expression of GLT-1c in retinal ganglion cells [36]. The induction of GLT-1c expression by retinal ganglion cells may indicate that the perturbation in glutamate homeostasis is evident in glaucoma and that such anomalies selectively influence retinal ganglion cells. These results suggest that the expression of GLT-1c may represent an attempt by retinal ganglion cells to protect themselves against elevated levels of glutamate. GLT-1c may be a useful indicator of the extent of stress of the retinal ganglion cells and thus a tool for examining outcomes of potential therapeutic and experimental interventions.

*EAAC-1*. Harada et al. [29] utilized EAAC1-deficient mice and examined the long-term effect of retinal morphology during postnatal development on RGC survival *in vivo*. They found that EAAC-1-knockout mice showed spontaneously occurring RGC death and typical glaucomatous damage of the optic nerve without elevated IOP. The main role of EAAC-1 is to transport cysteine into RGCs as a precursor for neuronal glutathione synthesis [47, 110–112]. Thus, EAAC-1 deficiency induces RGC loss mainly through oxidative stress.

#### 3.2.2. Glutamine Synthetase

It remains controversial whether pressure elevation changes the expression and activities of glutamine synthetase. Although increases in the expression of glutamine synthetase were reported after pressure elevation [21, 113, 114], decreases in activity and expression of glutamine synthetase have been also reported [115, 116]. Shen et al. [113] reported that high concentration of vitreal glutamate induced upregulation of glutamine synthetase. Such upregulation was blocked if IOP was acutely elevated for 24 hours, but was restored if IOP remained elevated for 1 week.

These findings suggest that moderate elevation of IOP causes only short-term functional changes of glutamate metabolism by retinal Müller cells. However, it is not known to what extent endogenous extracellular glutamate can regulate glutamine synthetase expression in normal eyes or in eyes with glaucoma.

In primary glaucoma in dogs [116], it is reported that decreases in glutamine synthetase immunoreactivity were associated with the damaged regions of the retina. These findings may indicate that the decrease in glutamine synthetase potentiates ischemia-induced early glutamate redistribution and neuronal damage in canine primary glaucoma.

Ishikawa et al. examined the enzyme activity [104] and expressional changes of GLAST [117] induced by elevated pressure (75 mmHg) for 24 hours using a rat *ex vivo* hydropressure model. In this acute model, elevated pressure suppresses activity and expression of glutamine synthetase. In addition, it is revealed that depressed GLAST expression results in downregulation of glutamine synthetase activity. These results may indicate that during pressure-loading, impairment of GLAST takes place first, and results in downregulation of glutamine synthetase activity as a second effect.

#### 3.2.3. Energy Deprivation

It has been reported that the RGC loses the retrograde axonal transport by mechanical causes, resulting in apoptotic or necrotic cell death in the experimental glaucoma model induced by perilimbal and episcleral vein photocauterization [118]. Axonal transport is the prominent energy-consuming process to transport cell organelles, neurotrophic factors, and other substances [119]. Ju et al. [120] reported that IOP elevation directly damaged mitochondria in the optic nerve head axons in the glaucomatous DBA/2J mice. Mitochondrial impairment induces cellular ATP reduction, resulting in disturbance of axonal transport and influence the viability of the retinal ganglion cells via retrograde axonal degeneration [121, 122]. Excitotoxicity is also considered to be closely associated with ATP-depletion induced by mitochondrial dysfunction [123–128]. These results indicate the important features about the potential risk of energy deprivation in the glaucoma.

### 3.3. Diabetic Retinopathy

Diabetic retinopathy is the most common complication of the diabetes, and a leading cause of visual impairment during the working-age in the industrialized countries [129]. Diabetic retinopathy has been classically considered as a microcirculatory disease of the retina. However, it has been recently reported that retinal degeneration precedes the impairment of the microcirculation. In the early stage of the diabetic retinopathy, elevated levels of glutamate, oxidative stress, the overexpression of the renin-angiotensin system, and the upregulation of receptor for advanced glycation end-products play an essential role [130]. These results mean that main features of retinal degeneration are already present in the retinas of diabetes without any microcirculatory abnormalities. In the later stage, retinal excitotoxicity participates in vascular endothelial growth factor (VEGF-) induced microcirculatory abnormalities such as disruption of blood-retinal barrier or an increase in the vascular permeability.

The relationship between the excitotoxicity and the induction of VEGF is one of the most interesting pathways linking neurodegeneration with vascular impairment. NMDA receptors exert a tonic inhibition of VEGF secretion in cultures of rat purified Müller cells, thus indicating that in healthy retina glutamatergic, stimulation could have a protective role [131]. In addition, it has been demonstrated that the elevation of VEGF expression and blood-retinal barrier (BRB) breakdown in streptozotocine-induced diabetic rats are blocked by the NMDA receptor channel blocker and the uncompetitive antagonist memantine [132].

These results suggest that hyperglycemia induces an increase in extracellular glutamate and the subsequent over-activation of NMDA receptors mediates VEGF production, BRB breakdown, and RGC damage observed in diabetic retinopathy. In this regard, it has recently been reported that the attenuation of retinal NMDA receptor activity by brimonidine (an alpha-2 adrenergic receptor agonist) results in a marked decrease in vitreoretinal VEGF and the inhibition of BRB breakdown in diabetic rats [132].

#### 3.3.1. Glutamate Transporters

Increased concentration of glutamate in vitreous body of diabetic retinopathy patients has been reported [133]. Early in the course of diabetic retinopathy, the function of the glutamate transporter in Müller cells is reported to decrease by a mechanism that is likely to involve oxidation [134].

As the activity of glutamate transporter can be rapidly restored, it seems possible that targeting this molecule for therapeutic intervention may restore glutamate homeostasis, and ameliorate sight-threatening complications of diabetic retinopathy [135].

Recently, Lau et al. [136] reported decreases in the transcript levels of genes related to glutamate neurotransmission and transport as diabetes progresses in the rat retina. Diabetes caused significant decrease in the transcriptional expression of glutamate transporter *SLC1A3* gene encoding GLAST protein, leading to the decreased removal of glutamate from the extracellular space, suggesting that diabetes impairs the glutamate transporter function of Müller cells. Consistently, faint GLAST immunoreactivity was restricted to the inner retina with the diabetic retinopathy compared with the throughout staining of the normal retina [137].

#### 3.3.2. Glutamine Synthetase

In diabetic rats, an increase in glutamine synthetase and a decrease in glutamate transporter are reported [138].Series Analysis of Gene Expression (SAGE) analysis of the diabetic retina revealed a 45.6% reduction in transcript levels of glutamine synthetase in streptozotocin-induced diabetic rats compared with normal rats [139]. RT-PCR and colorimetric enzyme activity assays revealed significant decreases in glutamine synthetase mRNA expression and the enzyme activity as early as the first month of diabetes development, with a progressive decrease in GS mRNA level and enzyme activity over a 12-month period. Northern blot analysis indicated a linear correlation between the reduction in glutamate synthetase expression and the time course of diabetic retinopathy, which was validated by real-time RT-PCR. These results implicate glutamine synthetase as a possible biomarker for evaluating the severity of developed diabetic retinopathy over the time course of diabetes progression. Immunohistochemistry revealed that antiglutamine synthetase labeling was prominent in the outer and inner plexiform layer as well as the ganglion cell layer in the normal control, while the signal was diminished in the diabetic retina compared to control retina [137]. By contrast, Silva et al. reported that Müller cells exposed to high-glucose medium produced higher levels of glutamine synthetase, but reduced levels of glutamate transporter [138].

## 4. Concluding Comments

Glutamate uptake and its enzymatic degradation are the critical steps to maintain the homeostasis of extracellular glutamate concentration in the retina. Down-regulation of these process induces excitotoxic neuronal degeneration in retinal diseases such as ischemia, glaucoma, and diabetic retinopathy. The abnormalities in glutamate metabolism are also caused by energy failure due to mitochondrial dysfunction. As glutamine synthetase consumes ATP to convert glutamate to glutamine, ATP depletion induces the impairment of delegation of glutamate, resulting in the elevation of the extracellular concentration of glutamate. Although glutamate transporters do not require ATP consumption, they are the Na^+^-dependent glutamate transporters, which utilize ionic gradients of Na^+^, K^+^, and H^+^ to drive glutamate transport against the concentration gradient. Since the ionic gradients are maintained by the sodium pump, they are dependent upon ATP production. When ATP levels drop, both glutamate uptake and degradation are remarkably inhibited, and the risk to induce excitotoxicity significantly increases.

Retinal ischemia induces irreversible excitotoxic degeneration as the consequence of ATP depletion. Reperfusion after ischemia induces exaggerated retinal degeneration. In ischemia/reperfusion model, GLAST function decreases in the early phase, followed by prompt functional recovery. Glutamine synthetase activity is upregulated for a short period after onset of ischemia to protect retinal neurons against excitotoxicity. As the rates of glycolysis decrease according to the depletion of glycogen storage, glutamine synthetase activity decreases. In the experimental glaucoma model, elevated IOP suppresses GLAST expression first, and results in the downregulation of glutamine synthetase activity as a second effect. IOP elevation also damaged mitochondria, inducing cellular ATP depletion, resulting in ganglion cell death. In the diabetic rat, the decrease in the expression of glutamate transporters and glutamine synthetase is reported. Hyperglycemia also induces an glutamate excess, and the subsequent overactivation of NMDA receptors mediates VEGF production and RGC damage.

These results indicate the importance of maintaining the homeostasis of glutamate metabolism to prevent the excitotoxicity in retinal diseases such as the retinal ischemia, glaucoma, and diabetic retinopathy.
